# Comprehensive training load monitoring with biomarkers, performance testing, local positioning data, and questionnaires - first results from elite youth soccer

**DOI:** 10.3389/fphys.2022.1000898

**Published:** 2022-10-03

**Authors:** Nils Haller, Julia C. Blumkaitis, Tilmann Strepp, Anna Schmuttermair, Lorenz Aglas, Perikles Simon, Elmo Neuberger, Christina Kranzinger, Stefan Kranzinger, James O’Brien, Bernd Ergoth, Stefan Raffetseder, Christian Fail, Manfred Düring, Thomas Stöggl

**Affiliations:** ^1^ Department of Sport and Exercise Science, University of Salzburg, Salzburg, Austria; ^2^ Department of Sports Medicine, Rehabilitation and Disease Prevention, University of Mainz, Mainz, Germany; ^3^ Red Bull Athlete Performance Center, Salzburg, Austria; ^4^ Department of Biosciences and Medical Biology, University of Salzburg, Salzburg, Austria; ^5^ Salzburg Research Forschungsgesellschaft M.B.H, Salzburg, Austria

**Keywords:** football (soccer), training load, monitoring, performance, injury prevenition

## Abstract

Load management, i.e., prescribing, monitoring, and adjusting training load, is primarily aimed at preventing injury and maximizing performance. The search for objective monitoring tools to assess the external and internal load of athletes is of great interest for sports science research. In this 4-week pilot study, we assessed the feasibility and acceptance of an extensive monitoring approach using biomarkers, neuromuscular performance, and questionnaires in an elite youth soccer setting. Eight male players (mean ± SD: age: 17.0 ± 0.6 years, weight: 69.6 ± 8.2 kg, height: 177 ± 7 cm, VO_2max_: 62.2 ± 3.8 ml/min/kg) were monitored with a local positioning system (e.g., distance covered, sprints), biomarkers (cell-free DNA, creatine kinase), questionnaires, neuromuscular performance testing (counter-movement jump) and further strength testing (Nordic hamstring exercise, hip abduction and adduction). Feasibility was high with no substantial impact on the training routine and no adverse events such as injuries during monitoring. Adherence to the performance tests was high, but adherence to the daily questionnaires was low, and decreased across the study period. Occasional significant correlations were observed between questionnaire scores and training load data, as well as between questionnaire scores and neuromuscular performance. However, due to the small sample size, these findings should be treated with caution. These preliminary results highlight the feasibility of the approach in elite soccer, but also indicate that modifications are needed in further large-scale studies, particularly in relation to the length of the questionnaire.

## 1 Introduction

Soccer usually leads to glycogen depletion, muscular and mental fatigue, and a performance decline over several days following a match (Ispirlidis et al., 2008; Nédélec et al., 2012). Subsequent recovery time is influenced by multiple factors, including playing/training time-position, endurance performance and, or metabolic predispositions (Mohr et al., 2003; Nédélec et al., 2012; Marqués-Jiménez et al., 2017). Modern soccer has evolved considerably, as evidenced by an increase in total distance and high-intensity actions, along with lower work-to-rest ratios (Barnes et al., 2014; Wallace and Norton, 2014). In recent times, congested competition schedules have resulted in high workloads, especially for elite teams and their best players. In particular, spikes in workload have been identified as a contributing factor to injury, fatigue, and performance decline (Gabbett et al., 2014; Gabbett, 2020).

The increased demands in professional soccer illustrate the need for adequate load management (i.e., the prescription, monitoring, and adjustment of workload (Soligard et al., 2016)). Inadequate prescription of training load and recovery can lead to performance decline, overtraining, or injury. It is thus crucial to monitor the external (“the prescribed physical work”) and internal (“the psychophysiological response”) aspects of training load to understand athlete fatigue, assess recovery time, and detect a decline in performance early. The overall goal is to individualize load and recovery to optimize performance and reduce the risk of injury (Halson, 2014; Spörri et al., 2017; Impellizzeri et al., 2019).

To date, however, there is no gold standard for monitoring player load (Halson, 2014; Impellizzeri et al., 2019). External load is typically measured with global positioning satellite systems, local positioning systems (LPS) or video-camera-based systems (Pettersen et al., 2018). For internal load, fatigue and recovery monitoring, questionnaires filled out by coaches (external assessment) or players (self-rating) are commonly used (Saw et al., 2017). However, athletes may perceive training sessions to be much harder than planned by practitioners, which can lead to long-term maladaptation (Brink et al., 2014). Another aspect is that players may intentionally underestimate their fatigue level to increase the probability of being nominated for the next game. Thus, it is recommended to combine subjective and objective monitoring tools (Halson, 2014; Marqués-Jiménez et al., 2017).

In this respect, a variety of objective monitoring strategies have been proposed, including e.g., the combination of lactate and heart rate (Coutts et al., 2009). In addition, monitoring neuromuscular performance variables of a countermovement jump (CMJ) (e.g., average jump height, peak power, mean power, peak velocity) may reflect aspects of athlete fatigue (i.e., “the inability to maintain performance at the required level”) (Claudino et al., 2017). However, further research is required to identify the optimal variables for monitoring the neuromuscular status (Gathercole R. J. et al., 2015), and performance assessments may induce additional fatigue in athletes. In this regard, biomarkers are another promising option for load and recovery monitoring. Blood-based parameters such as interleukins, creatine kinase (CK), C-reactive protein, or cell-free DNA (cfDNA) have shown promising increases in soccer settings at rest or under acute conditions (Ispirlidis et al., 2008; Thorpe and Sunderland, 2012; Haller et al., 2019). In particular, a significant correlation was shown for CK and sprint performance (Thorpe and Sunderland, 2012), as well as for cfDNA and total distance covered in a soccer game (Haller et al., 2018). Ispirlidis and others (Ispirlidis et al., 2008) demonstrated a pronounced inflammatory response after a soccer match, along with a performance decline over 3 days. These results highlight the potential of blood-based biomarkers for objective load monitoring. However, no single biomarker was exclusively able to cover all aspects of player load or recovery status (Halson, 2014; Marqués-Jiménez et al., 2017) or served as a predictor of increased risk of injury. A finding that is not surprising given the complex profile of intermittent exercise leading to a variety of physiological responses. This highlights the need to further observe biomarker panels in applied settings as well as the need for comprehensive monitoring approaches to reflect acute and chronic responses and adaptations to ultimately detect a decline in performance and impending injury early.

In this 4-week pilot study, we are testing the feasibility, and acceptance of an extensive monitoring approach using traditional and innovative biomarkers, performance assessments, and subjective questionnaires with the secondary objective of determining the relationships of the variables. All assessments were integrated into the daily training routine in a time-saving and non-obtrusive manner. To our knowledge, this multidisciplinary and holistic approach has not yet been studied with the parameters examined here. The present study will reveal the feasibility of the approach and identify parameters suitable for further investigation.

## 2 Methods

### 2.1 Ethical approval

The local human Ethical Board in Salzburg (GZ 02/2021) agreed with the experimental setup. All procedures conformed with the standards of the Declaration of Helsinki of the World Medical Association. Participants received both oral and written information about the study writing and provided written consent.

### 2.2 Participants and setting

**FIGURE 1:**
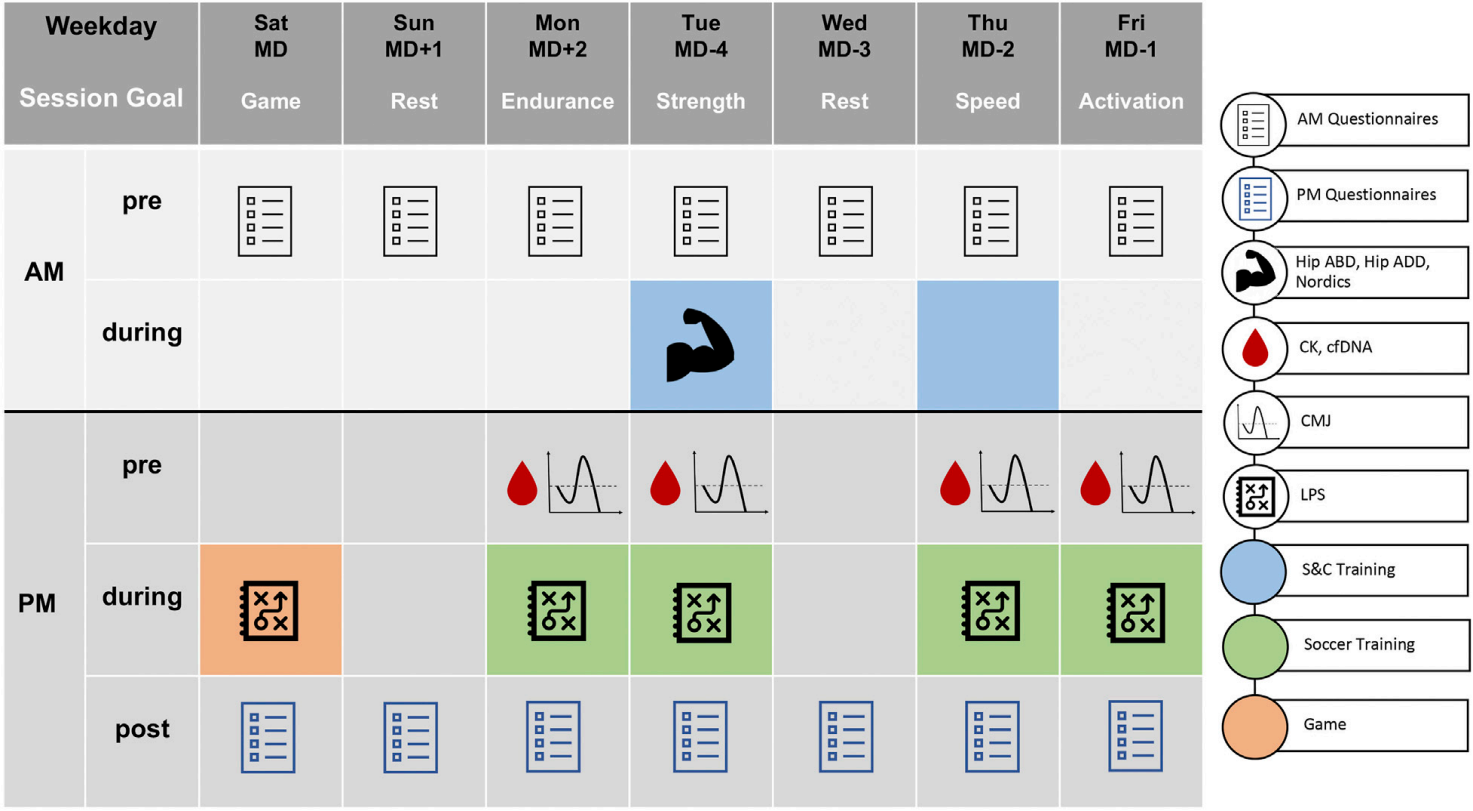
Outline of the monitoring approach. This exemplary week was repeated for all 4 weeks. The legend on the right illustrates which measures were performed on which day. MD = match day, AM = morning, PM = afternoon/evening, pre = before training, during = during training, post = after training, Hip ABD = hip abduction, Hip ADD = hip adduction, Nordics = Nordic hamstring exercise, CK = creatine kinase, cfDNA = cell-free DNA, CMJ = counter-movement jump, LPS = local positioning system (external and internal training load), S&C = strength and conditioning, “+”/“-” signs clarify the distance in days to regular season games.

Data from eight male players (of a 24-player roster, selected by the coaching staff; mean ± SD: age: 17.0 ± 0.6 years, weight: 69.6 ± 8.2 kg, height: 177 ± 7 cm, VO_2max_: 62.2 ± 3.8 ml/min/kg) of an elite European youth soccer team (first national league, participant of the UEFA Youth League) were collected during a 4-week period of the regular season in 2021. Researchers had no influence on the training program and no feedback was given to the coaching staff about preliminary results before the study was completed. Figure 1 outlines the study design.

A standardized setting with fixed test modalities was used every week to ensure a uniform procedure and comparability of measurements. Likewise, the training focus and number of training sessions per day were identical in each of the 4 weeks.

In detail, participants were asked to complete a questionnaire every morning and every evening. Strength and conditioning (S&C) training took place in the morning on 2 days of the week, with performance testing within the S&C training session on match day (MD) -4 (days). In the afternoon, blood sampling took place on 4 days during the week (MD +2, -4, -2, -1) before training under resting conditions, followed by measurement of counter-movement jump (CMJ) performance. These assessments were incorporated into the regular training session and took 15–20 min to complete. Performance testing, blood sampling as well as questionnaires were part of the training process prior to the study start. Thus, all participants were familiarized with these procedures. Team training (except S&C training) and matches were consistently monitored using an LPS.

### 2.3 Measures

#### 2.3.1 Performance & injury

Performance data (e.g., distance covered, heart rate, high metabolic power distance (HMPD), training impulse (TRIMP) (Stagno et al., 2007), total amount of sprints, accelerations, decelerations) was recorded with a 100 Hz LPS (Kinexon Precision Technologies, Munich, Germany) during games and training. Injury statistics were documented daily by the team physiotherapist, in accordance with established guidelines (Fuller et al., 2006).

#### 2.3.2 Questionnaires

Customized questionnaires designed with the help of our psychologists were filled out online via a Smartphone application using the Trayn software (Trayn, Sunnyvale, CA, United States) and administered daily with eight items in the morning and 23 items in the evening. Participants received regular push messages to fill out the questionnaires. Items were related to sleep (Thornton et al., 2017), well-being, muscular fatigue, and pain (Micklewright et al., 2017) as well as subjective perceived performance capability in the morning (AM; on a Likert Scale from 0 to 10, coded from 0 = *e*.g., very fatigued, very poor to 10 = *e*.g., no pain, very good *etc.*). Evening questions (PM) were related to previous training, stress enjoyment, pressure, mental performance, mood, and performance capability (Haken and Schiepek, 2010; Schiepek et al., 2018) (combination of visual-analogue-scales from 0 to 100 mm and Likert Scales from 0 to 10). For the complete questionnaire, please refer to Supplement Material S1.

#### 2.3.3 Nordic hamstring exercise

The Nordic hamstring exercise was integrated into the S&C training routine. Eccentric hamstring strength was measured on the Nordbord device (Vald Performance, Albion, Australia) on MD-4 (Figure 1) (Opar et al., 2013). Players knelt on the Nordbord with the fixation hooks orientated vertically and positioned just above their ankles (standardized position). Three repetitions with maximal effort and 5 s breaks were performed. The tester instructed players to keep their body straight and resist falling as long as possible. Verbal encouragement in the form of, „hold, hold, hold“ was given. The mean of the left and right leg maximal force (F_max_) of each trial entered the statistical analysis.

#### 2.3.4 Hip abduction, hip adduction

Isometric hip abduction and hip adduction was integrated into the S&C training. Isometric hip abduction and hip adduction strength were measured using the Groinbar device (Vald Performance, Albion, Australia) on MD-4 (Figure 1) (Ryan et al., 2019). Players were barefoot in the supine position, with their arms crossed in front of the chest. The hips were positioned in 45° flexion and neutral rotation. The medial femoral epicondyles were centered over the inner load cells for adduction, and the lateral femoral epicondyles over the outer load cells to test abduction. Following two warm-up repetitions at 50% and 75% of their maximum effort, players performed three repetitions at 100% effort for both movements. Each repetition was held for 5 s, with a 10 s break between repetitions. Verbal encouragement in the form of, 3, 2, one push, push, push“ was given. The warm-up and all three test repetitions for hip adduction were performed before hip abduction. The mean of left and right leg F_max_ of each trial entered the statistical analysis.

#### 2.3.5 Neuromuscular performance

Neuromuscular performance was measured *via* jump performance during the CMJ on MD +2, -4, -2, -1 (Figure 1). The CMJ was performed on two separate force plates (Forcedecks, VALD Performance, Albion, Australia), with arms fixed at the hip. Prior to the jumps, the athletes performed a standardized warm up (10 min treadmill running at 8 km/h). Following two warm-up jumps, three maximal jump attempts were performed in a standardized order (Twist and Highton, 2013; Gathercole R. J. et al., 2015; Watkins et al., 2017). Participants were instructed to jump as high as possible in each trial. The depth of the CMJ was chosen by the participants themselves.

#### 2.3.6 Blood collection

Blood collection took place on MD +2, -4, -2, -1 at rest prior to the CMJ and the training in the afternoon. For measurement of CK concentrations, 32 μL of capillary blood was drawn from the fingertip and analyzed immediately after collection *via* the Reflotron Sprint system (Roche Diagnostics, Basel, Switzerland) at a standardized time before training. For analysis of cfDNA concentrations, approximately 10–15 μL of capillary blood was drawn from the fingertip and centrifuged immediately at 1600 x g for 10 min. Plasma was then stored at < −20°C. cfDNA was quantified by analyzing unpurified plasma *via* quantitative polymerase chain reaction (qPCR) as described elsewhere (Neuberger et al., 2021). In short, plasma was diluted 1:10 in H_2_O as a template for qPCR, which was based on the amplification of a 90-base pair sequence located inside the L1PA2 transposon. A CFX384 Touch™ real-time PCR system (Bio-Rad, Munich, Germany) was used for the analysis of blood samples using the following protocol: denaturation at 98°C for 2 min, 35 cycles of melting at 95°C for 10 s, annealing at 64°C for 10 s, followed by a melting curve (Neuberger et al., 2021).

### 2.4 Statistical analysis

Data were collected using Microsoft Excel 2016 (Microsoft Corp, Redmond, WA, United States of America), and statistical analysis was performed using JMP14 (SAS Inc., Cary, NC, United States of America) and R version 4.1.0 (R: A Language and Environment for Statistical Computing, Vienna, Austria). Feasibility was determined by the number of adverse events and dropouts. Acceptability was determined through adherence, which was calculated according to the following formula: number of tests or questionnaires performed: total number of planned tests or questionnaires. In addition, we provide an exploratory analysis of selected parameters. Seven parameters (cfDNA, CK, CMJ jump height, perceived recovery, perceived sleep quality, HMPD, TRIMP) were visualized over the 4-week period to show the evolution of these parameters over time. To identify relationships between the variables, correlation analysis was performed for the total group. Due to the nature of the data (ordinal and continuous), the Spearman rank coefficient was calculated. The alpha-level was set to 0.05. Depending on the test modalities (see Figure 1), the variables were correlated with the data of the same or the following day (day 1 or 2). Finally, to identify possible redundant questions and to shorten the questionnaire for future studies, a hierarchical cluster analysis was performed based on the ICLUST algorithm (Revelle, 1978) implemented in the R package psych (Revelle, 2020). For the visualizations the R-packages ggplot2 (Wickham, 2016) and ggcorplot (Erichson, 2021) were used.

## 3 Results

**FIGURE 2:**
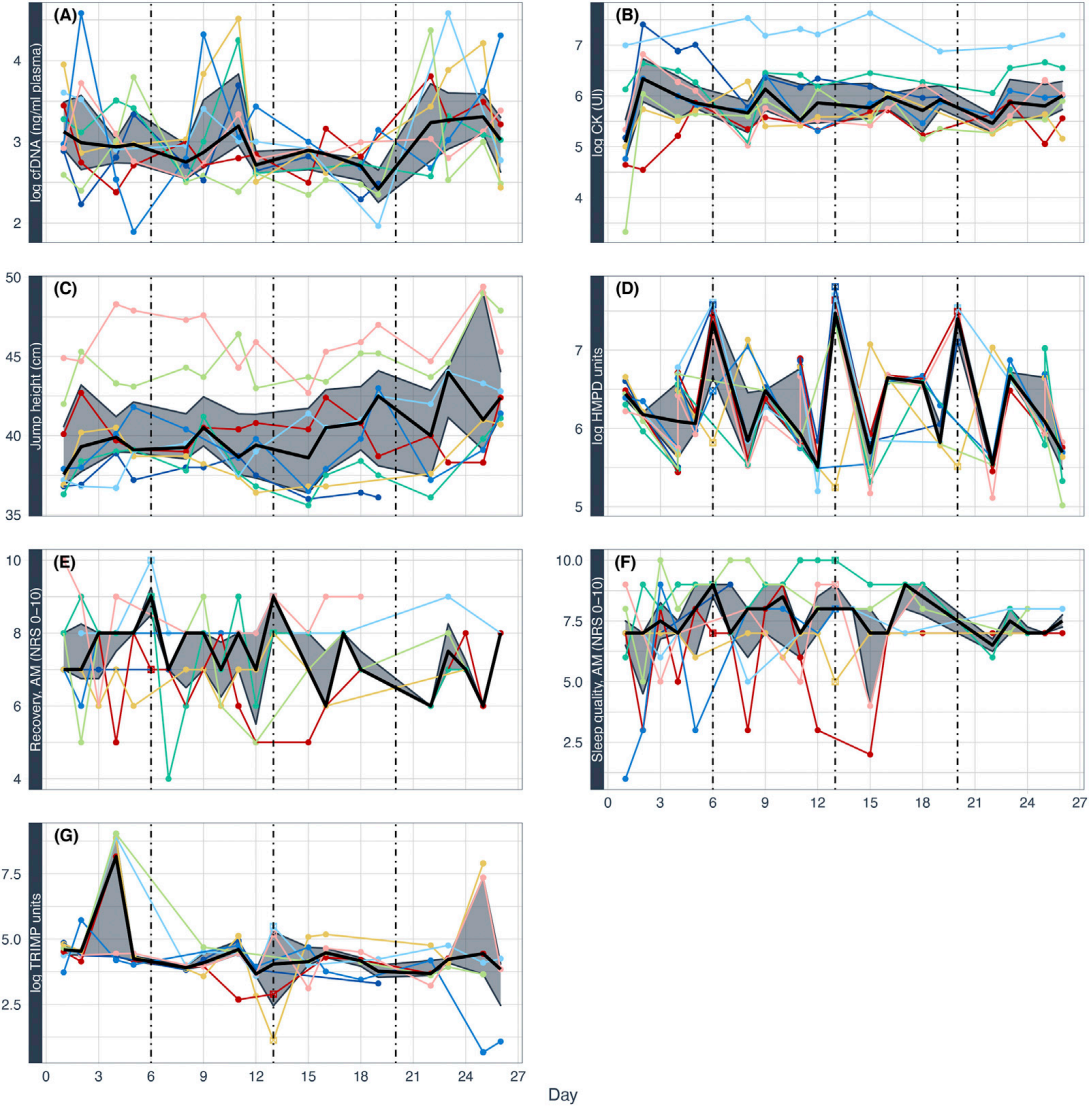
Selected variables over the course of 4 weeks in all eight players. cfDNA (cell-free DNA) **(A)** and CK (creatine kinase) **(B)**, countermovement jump height **(C)**, HMPD (high metabolic power distance) **(D)**, perceived recovery **(E)**, sleep quality **(F)**, and training impulse (TRIMP) **(G)**. For each plot, each player is visualized with the same color. The black line displays the median of the variable, the grey ribbon the interquartile ranges. Dashed lines represent games on days 6, 13, and 20. cfDNA, CK, TRIMP, and HMPD log(logarithmized)-transformed, jump height in cm and questionnaire scores on a scale from 0 to 10. Log HMPD on days six and 20 could only be calculated by averaging the individual available data for each match period due to the lack of data from the LPS provider.

Figure 2 provides a descriptive view of the changes in seven selected variables over time across all eight players. During the study period, three games and 19 soccer training sessions took place. Here, it is already evident that the variables show strong inter- and intraindividual differences. At first glance, the variables presented do not follow a consistent pattern, however, spikes in the HMPD variable are evident (days 6, 13, 20) corresponding with season games.

### 3.1 Aspects of feasibility and acceptance

There were no dropouts or adverse events during the 4-week study period related to the monitoring. Capillary sampling and all performance testing procedures were well accepted by players. Missing data (also described as percentages calculated through the formula missing values: total assessments) were 12/128 = 9% for CMJ, 8/128 = 6%, for CK, 7/128 = 5% for cfDNA; 4/32 = 13% for Nordic hamstring exercise, 2/32 = 6% for hip adduction; and 3/32 = 9% for hip abduction, respectively. These missing data resulted from the fact that the players were not available due to injury (non-contact injury n = 1; contact injuries n = 2 players) or due to pain before or during the Nordic hamstring or hip adduction/abduction exercises. In two other cases, CK was not measurable even after dilution of the sample. One player stopped the Nordic hamstring exercise after two attempts and two players did not perform all repetitions of the hip abduction and adduction exercise due to the onset of perceived pain. In these cases, the team physiotherapist decided to stop the testing to lower the risk injury. Regarding subjective measures, the AM questionnaires were filled out during week one in 45 (i.e., 45/56 = 80%) cases and declined to 36 (64%), 17 (30%), and 13 (23%) cases, at weeks 2–4, respectively. Likewise, the PM questionnaire was completed in 38 (68%) cases in week 1, but in only 24 (43%), 9 (16%), and 8 (14%) cases, respectively, at weeks two–4. Coaches did not express any complaints regarding the extra effort and time required regarding the additional testing prior to training (15–20 min for blood sampling, and CMJ).

**FIGURE 3:**
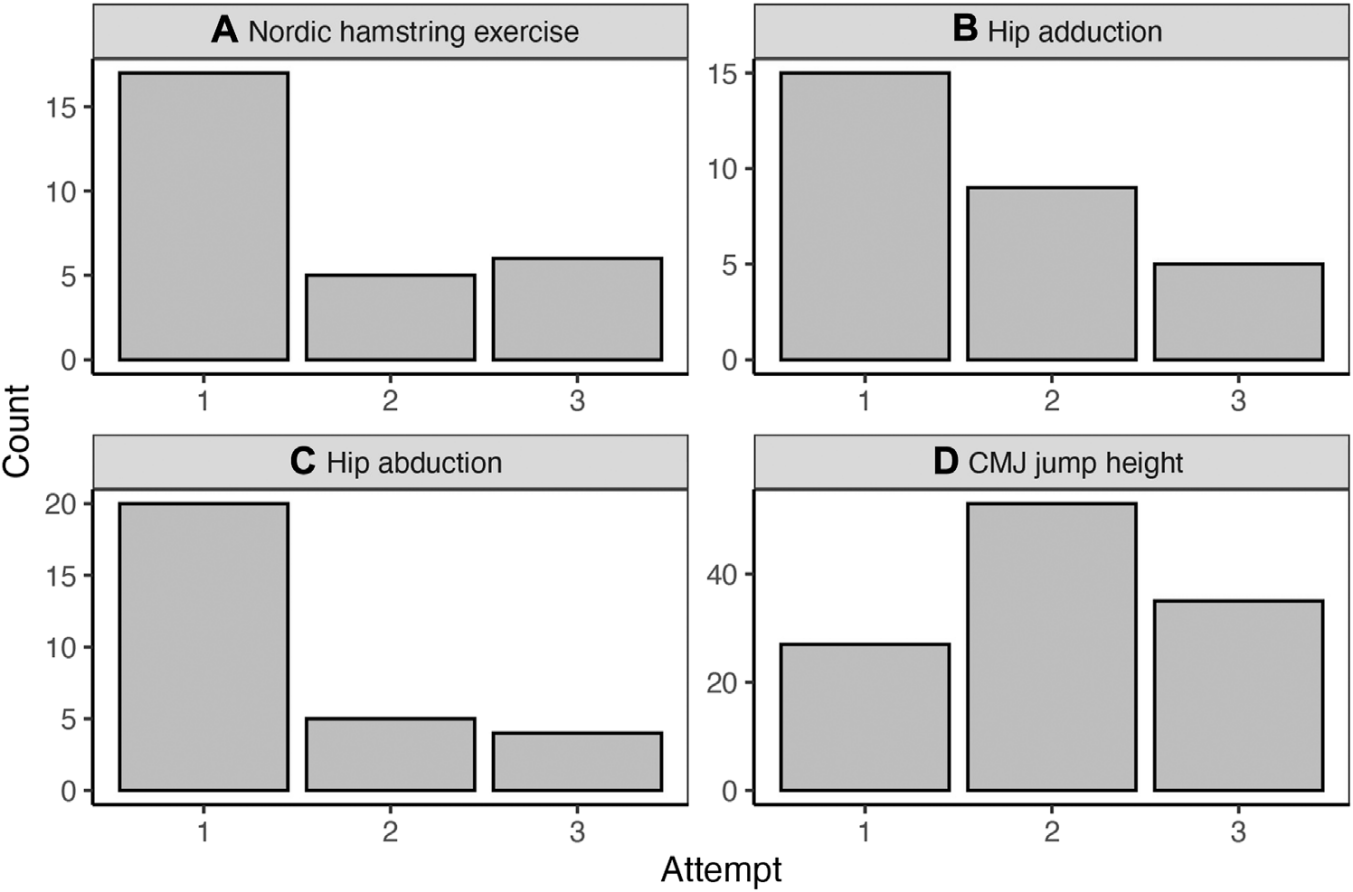
Descriptive representation of the best attempt (number of cases on the *y*-axis) in each test, i.e., Nordic hamstring exercise, hip adduction, hip abduction and countermovement jump performance **(A)** Nordic hamstring exercise. **(B)** Hip adduction **(C)** Hip abduction **(D)** CMJ jump height.

Figure 3 shows the best performance out of the three trials of each assessment. While CMJ performance was highly variable and most frequently highest on the second attempt, athletes’ Nordic hamstring, hip adduction and abduction strength were most frequently best on the first attempt.

### 3.2 Correlation analysis

**FIGURE 4:**
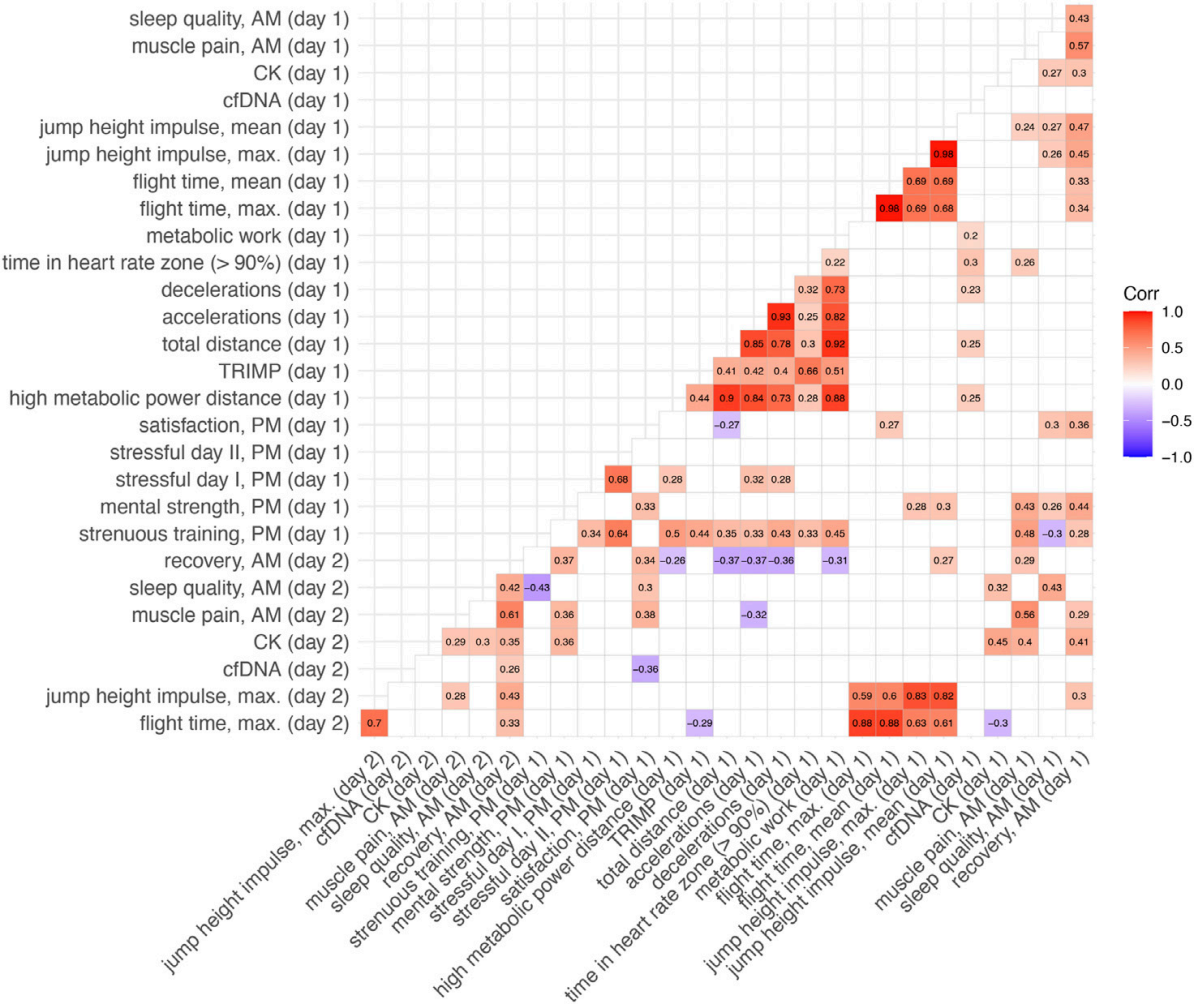
Correlation matrix (spearman rank correlation) with selected performance variables, questionnaire data and biomarker concentrations. We hypothesize that questionnaire results (recovery, sleep, muscle pain) and countermovement jump data collected each morning, as well as biomarkers collected in the afternoon, may be influenced by the previous day’s training load. Therefore, these seven variables (marked as “day 2”) were correlated with the training data of the previous day (“day 1”). AM = morning, PM = evening, cfDNA = cell-free DNA, CK = creatine kinase, TRIMP = training impulse. Non-significant correlations are omitted from this figure; see Supplementary Material S3 for a summary of all correlations. The following questionnaire items from the supplementary material were selected: 1., 6., and 8 (AM questionnaire); 1., 2., 3., and 5. (PM questionnaire).

The following correlation matrix (Figure 4) shows the pairwise correlations between the various variables obtained during the study. The most striking correlations were found between the different tracking variables (HMPD, total distance, decelerations, accelerations). In addition, significant correlations were found between questionnaire variables as well as between some of the questionnaire variables and the performance variables (e.g., CMJ performance and perceived recovery AM and sleep quality AM; HMPD, total distance with strenuous training PM). There was a near perfect correlation between maximal vs. mean jump height (r = 0.98), while the correlation between the calculation methods (flight time vs. force impulse) was rather moderate (r = 0.69). In contrast, no correlation was found between CK and cfDNA (Supplement Material S3). However, CK showed some significant correlations to questionnaire data, while cfDNA correlated with some of the training data variables.

As questionnaire adherence decreased over time, and due to the large number of questionnaire items (eight items AM, and 23 items PM), an item cluster analysis to identify related clusters from all questionnaire items was performed. The two questionnaires contained several manifest variables to infer a latent variable (e.g., the psychological stress). The goal was to reduce the item number in agreement with the psychologist, which may ultimately lead to higher adherence. The analysis specifies one large cluster for the AM items and two clusters for the PM items and can be found in the Supplement Material S2, indicating that certain questions show high consistency and could be removed from the respective cluster after detailed discussion with the responsible psychologist.

## 4 Discussion

The overall objective of this study was to test the feasibility and acceptability of a comprehensive monitoring approach using objective (neuromuscular performance, strength testing and biomarkers) and subjective (questionnaire) measures for training load, stress, and recovery in an elite youth soccer setting. Most of the applied measures were accepted by players (as determined by adherence) and all measures could be integrated into the daily routine in a time-efficient and minimal-invasive manner without influencing the training process (expressed by qualitative feedback from the coaches). These positive results could be attributed to the fact that the respective measures were already known prior to the start of the study and could be integrated into the training process with little time expenditure. New for the players and coaches were only the extensive questionnaires that had to be filled out outside the regular training sessions and the extensive standardized monitoring (e.g., CMJ and blood sampling four times per week). Despite players being reminded by the staff on a regular basis, questionnaire adherence decreased markedly during the intervention period.

It is well accepted that a successful integration of a holistic monitoring approach into the training regime depends on 1) coach buy-in, 2) costs, time, and logistical aspects, 3) team adherence; 4) an interdisciplinary team and 5) coaching staff that see an added value of an empirical based measure (Akenhead and Nassis, 2016). Therefore, most of the measures were integrated into the daily training routine with the help of an interdisciplinary team of practitioners, scientists, and physicians, aiming not to disrupt the training process and to prevent additional burden on the players. In particular, a 15–20 min time period prior to the training was sufficient to perform capillary blood sampling (CK and cfDNA) and CMJ performance measurement. We mainly relied on simple testing assessments that do not require in-depth training by the athletes (e.g., CMJ, capillary blood sampling). Measures of hamstring and hip adductor and abductor strength was categorized as strength training stimulus and therefore integrated into the S&C training on MD-4. This performance testing did not lead to any adverse events.

Questionnaires have been shown to be a sensitive measure of changes in training load (Saw et al., 2016) and should thus be part of a holistic monitoring approach. In line with these findings, some significant negative correlations were observed between questionnaire scores and the tracking variables (e.g., between distance covered and perceived satisfaction PM and between distance covered and perceived recovery AM on the next day) further highlighting these instruments as reliable measures of training load and recovery. However, the adherence to the questionnaires declined over the study period which can be attributed to the large number and similarity of questions. These factors have previously been shown to be associated with poor adherence and data quality (Eisele et al., 2022). Of note, the number of items can be substantially reduced with identifying questionnaire clusters (Supplementary Material) which leads us to the assumption that a markedly reduced item number and, thus a more succinct questionnaire (Jenn, 2006), could lead to higher adherence in future studies (Eisele et al., 2022). In addition, an introductory and educational session (McGuigan et al., 2022) highlighting the added value for players (injury prevention, performance maximization) and thus increasing player understanding of the monitoring process could further improve acceptance. We do also believe that the involvement of a subset of eight players may have led to motivational issues and missed cohesiveness within the team. Therefore, the entire team should be integrated in such a study in order to avoid within team dynamics. It should be noted that blood collection and the performance tests were integrated into the regular training schedule, while the questionnaires had to be filled out by the players themselves in the morning and in the evening, possibly leading to differences in adherence between the monitoring tools.

Besides correlations between performance variables and questionnaire data, significant correlations of biomarker concentrations with further variables were observed. In particular, weak significant correlations between CK and muscle pain as well as perceived recovery were found. This underlines the potential of CK to objectively reflect aspects of fatigue and recovery needs which is consistent with previous findings (Mäestu et al., 2006; Sikorski et al., 2013; Berriel et al., 2020). Surprisingly, cfDNA correlated positively with some performance variables, suggesting predictive potential for performance. This observation is somewhat surprising given that previous studies only revealed an association with the previous training load in soccer (Haller et al., 2018; Haller et al., 2019) with one study showing even an association with overtraining in weightlifting athletes (Fatouros et al., 2006). However, due to the small sample size and the short study period, all correlations should be viewed with caution. It was not possible to perform individual analyses or more sophisticated statistical procedures such as survival analysis to predict a certain outcome. In addition, biomarkers are susceptible to confounding variables such as diet, or stress (Pedlar et al., 2019). Consequently, the choice of a standardized afternoon time point for our analyses and the integration of the examinations into the regular training may imply that unknown confounding variables, such as diet or prior physical activity, influenced the biomarker concentrations. In the case of cfDNA, for instance, psychological stress (Hummel et al., 2018), but also light physical activity (Haller et al., 2017) may have already increased cfDNA concentrations prior to sampling. This dilemma Buchheit (2014) described as “ideal vs. real”. The “ideal” situation would be a blood collection in the morning in a fasted state (Pedlar et al., 2019), while “real” would be to incorporate this into the training routine. However, we strongly recommend to move blood sampling in an upcoming study to the morning hours to widely exclude influencing factors and to allow meaningful interpretations of the blood concentrations (Pedlar et al., 2019). One may consider whether venous blood collection at less selected time points with a comparatively higher blood volume could lead to more meaningful results and allow for more sophisticated analysis such as proteomics approaches, or further DNA analysis (Moss et al., 2018; Contrepois et al., 2020). This would allow the identification of further useful biomarkers for load monitoring. However, venous blood sampling is not well accepted among elite athletes (Carling et al., 2018; Pedlar et al., 2019). Finally, additional information on biomarker suitability could be obtained if acute concentrations of load-sensitive biomarkers, such as cfDNA, were collected before and after the regular training sessions (Lee et al., 2017).

Lastly, it was demonstrated that CMJ, hip ab- and adduction, and Nordic hamstring exercise are appropriate for inclusion into the training regime in a time-efficient manner. This could potentially become even more efficient, as the first attempt of hip abduction, hip adduction, and Nordic hamstring performance, was frequently the best. A standardized protocol with one set of three maximal repetitions is most commonly performed (Opar et al., 2015; Timmins et al., 2016). In contrast, peak CMJ performance varied between repetitions, implying that multiple jumps are required. Average jump height of several repetitions compared to the best performance is potentially a more sensitive measure to detect fatigue in athletes (Al Haddad et al., 2015; Claudino et al., 2017). Further CMJ variables relating to movement quality could be affected in fatigued athletes. Thus, a CMJ-variable battery reflecting both output and movement strategy should be incorporated in the analysis in future studies (Gathercole R. et al., 2015). Interestingly, the moderate correlation between the calculation method of jump height (flight time vs. force impulse) suggests that confounding factors can affect the measurement result (e.g., weight measurement, landing position).

Based on the results of our pilot study, several recommendations for future studies can be made. To improve questionnaire adherence, the number of items should be markedly reduced combined with a pre-study educational lecture that highlights the benefits for the players. To obtain meaningful results and to allow for an individualized analysis, more data points are needed. The small sample size of players and short study period, in addition to occasional missing data made a more detailed and individualized statistical analysis difficult. Future studies with more participants and longer study periods could apply more in-depth analyses and even machine learning approaches to determine the underlying causes of injuries. It should be noted, however, that monitoring an entire soccer team with such a comprehensive approach requires good planning as well as the necessary infrastructure and personnel to remain efficient and consequently not interfere with the training process. Furthermore, cluster analysis or correspondence analysis could be used to find hidden data patterns. Time series analysis could be used to detect structural breaks in, for example, training load data to serve as an early warning system for injuries or overload. To gain further valid insights into biomarker responses, a standardized blood sampling in the morning hours and/or a pre-vs. post-exercise time series study design could provide a meaningful understanding under different conditions at rest and during exercise (Lee et al., 2017). To save time and prevent fatigue, practitioners may consider performing the Nordic hamstring, hip adduction, and hip abduction exercises with one clearly instructed “maximal” attempt, whereas the CMJ may require multiple attempts. Future studies could include an acceptance questionnaire to obtain feedback from players, such as why certain tasks were not met. Given the marked homogeneity within the metrics presented (tracking, questionnaires, CMJ variables), it is worth considering whether statistical approaches can be used to reduce such large data sets in future studies. Moderate associations between biomarkers (e.g., CK, cfDNA) and between questionnaire data and training load need to be tested in individualized analyses in larger data sets.

## Data Availability

The raw data supporting the conclusions of this article will be made available by the authors on reasonable request.
