# Supplementary material for: Comprehensive training load monitoring with biomarkers, performance testing, local positioning data, and questionnaires - first results from elite youth soccer
Source: Front Physiol. 2022 Oct 3;13:1000898. doi: 10.3389/fphys.2022.1000898 (PMC9573975; doi:10.3389/fphys.2022.1000898)
Supplement: Supplementary file 1 [file Table1.DOCX]

Supplementary Material

**Supplement 1.** Morning Questionnaires (AM) and Afternoon/ Evening Questionnaires (PM).

**Morning Questionnaire:**

Please select a number that correspond with your current feeling:

1. How well did you sleep last night?

0 1 2 3 4 5 6 7 8 9 10

Very good

Very poor

1. How many hours did you sleep last night?

______________

1. How do you estimate your physical performance for today?

0 1 2 3 4 5 6 7 8 9 10

Very good

Very poor

1. I am feeling…

0 1 2 3 4 5 6 7 8 9 10

Not focused

Very focused

1. I am feeling…

0 1 2 3 4 5 6 7 8 9 10

Very energetic

Very powerless

1. My recovery is….

0 1 2 3 4 5 6 7 8 9 10

Very good

Very poor

1. My muscles feel…

0 1 2 3 4 5 6 7 8 9 10

Very fatigued

Not fatigued at all

1. My muscle pain is…

0 1 2 3 4 5 6 7 8 9 10

No pain at all

Very intense

**Afternoon / Evening Questionnaire:**

Please select a number or draw a line that correspond with your current feeling:

1. How strenuous was today’s practice?

0 1 2 3 4 5 6 7 8 9 10

Maximal

Rest

1. How stressful did you find today compared to an average day?

Significant less stressful

Significant more stressful

1. How stressful did you find today’s school day compared to an average day?

Significant less stressful

Significant more stressful

1. How stressful did you find today’s practice/game day compared to an average day?

Significant less stressful

Significant more stressful

1. Today I was satisfied with myself

Not at all

Very

1. Today I have felt joy

Not at all

A lot

1. Today my mood was depressed

Not at all

Very

1. Today I experienced inner tension

Not at all

Very strongly

1. Today my self-confidence was

Equal to zero

Very strong

1. Today I was insecure

Not at all

Extremely

1. Today I felt pressure

Not at all

Very intense

1. Today I felt comfortable in my body

Not at all

Very

1. Due to physical discomfort, I could not perform in my sport today as usual

Does not apply at all

Applies completely

1. My physical condition is

Very poor

Very good

1. I am mentally strong

Not at all

Very strong

1. I can fully realize my potential

Not at all

Applies completely

1. My psychological condition is

Very poor

Very good

1. I am confident about my sport

Not at all

Very

1. I am fully capable of performing my sport

Not at all

Applies completely

1. I get tired very quickly in my sport

Not at all

Very fast

1. I feel exhausted from my sport

Not at all

Very exhausted

1. Success in my sport is important to me

Not at all

Very important

1. I am able to achieve my full potential in my sport

Not at all

Fully

**Supplement 2**. Cluster analysis of AM (A) and PM (B) questionnaires. Alpha represents the mean split half correlation also known as Cronbach’s alpha and beta the worst split half correlation, known as Revelle’s beta (see for both metrics (Revelle, 2020)). There is no consensus in the literature so far on what cut-off value should be used to interpret alpha as acceptable or sufficient. However, it is usually assumed that the value of alpha should be higher than 0.7 to reach a satisfactory level (Taber, 2018). Beta should be higher than 0.5 to show acceptable reliabilities (Revelle, 1979). With respect to the AM questionnaire, item 1 and 2 (C3), 4 and 5 (C1), 7 and 8 (C2) form sub-clusters with high internal consistency. For example, items 4 and 5 show high internal consistency (alpha=0.9), one item could be removed from the questionnaires. The PM clusters indicate a similar cluster formation, with high internal consistencies throughout a majority of clusters.

.
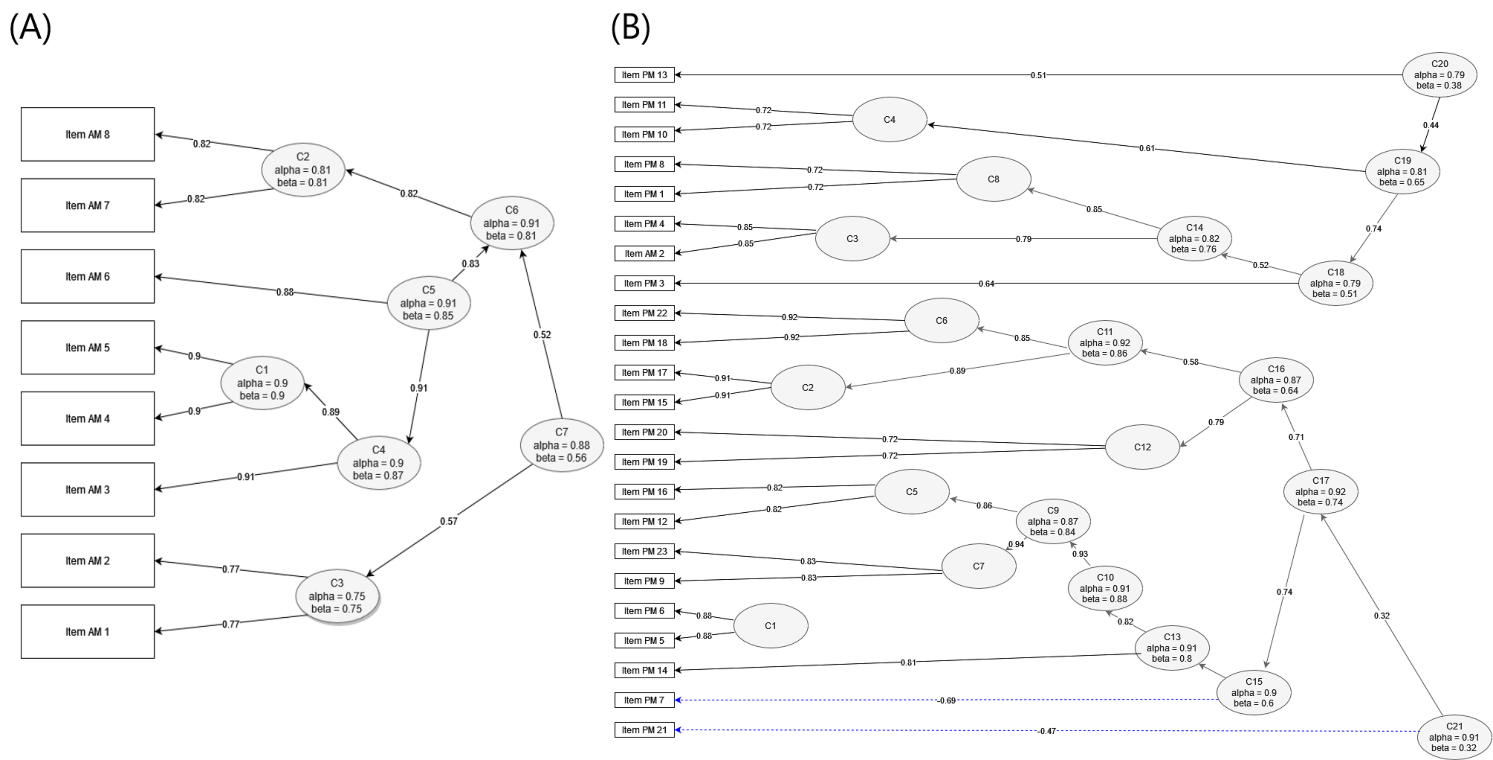


Taber, K.S. (2018). The Use of Cronbach’s Alpha When Developing and Reporting Research Instruments in Science Education. *Res. Sci. Educ.* 48(6)**,** 1273-1296. doi: 10.1007/s11165-016-9602-2.

Revelle, W. (1979). Hierarchical Cluster Analysis And The Internal Structure Of Tests. *Multivar. Behav. Res.* 14(1)**,** 57-74. doi: 10.1207/s15327906mbr1401_4.

Revelle, W. (2020). Psych: procedures for psychological, psychometric, and personality research. 2020. *R package version* 2(7).


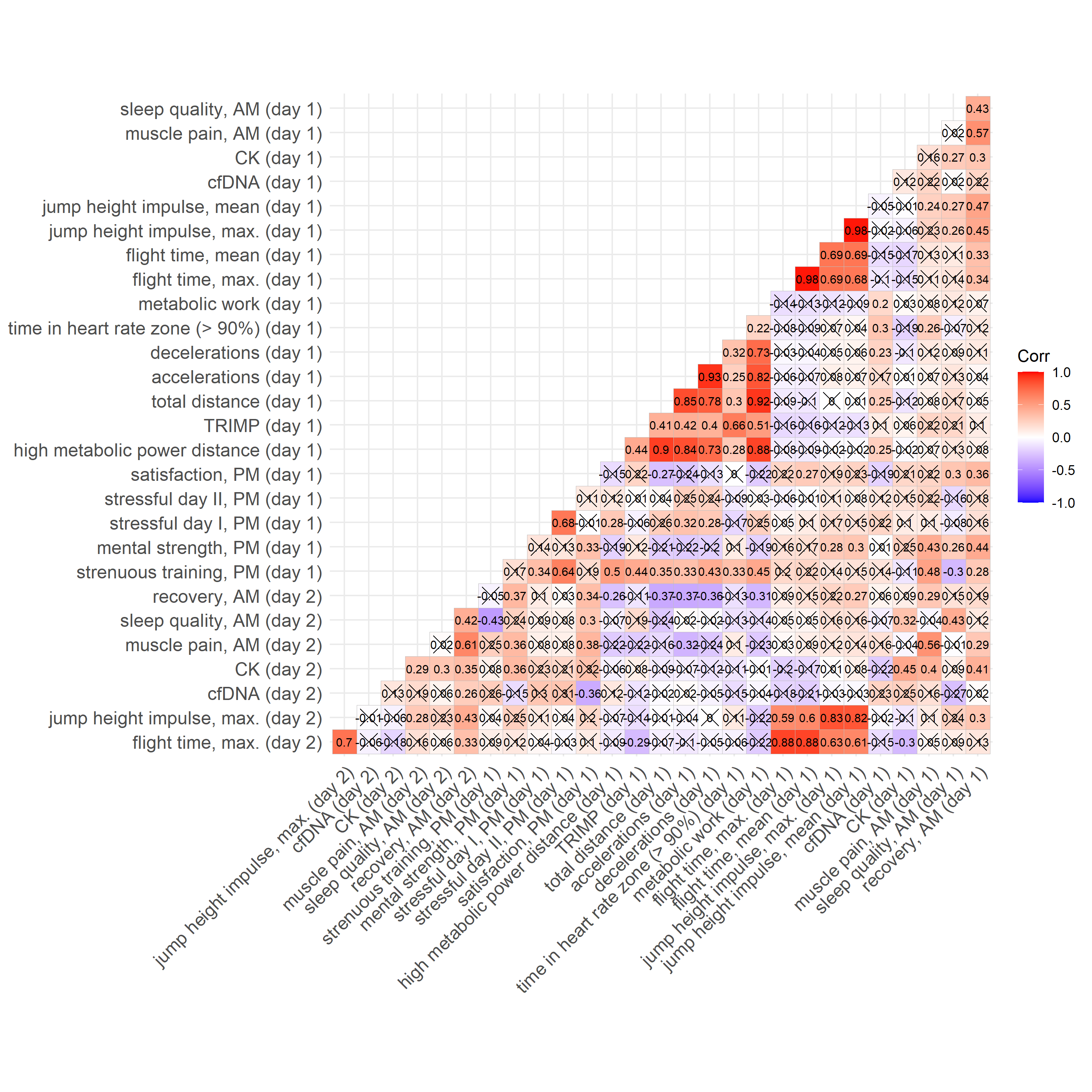


Supplement 3. Correlation matrix (spearman rank correlation) with selected performance variables, questionnaire data and biomarker concentrations. We hypothesize that questionnaire results (recovery, sleep, muscle pain) and countermovement jump data collected each morning, as well as biomarkers collected in the afternoon, may be influenced by the previous day's training load. Therefore, these 7 variables (marked as “day 2”) were correlated with the training data of the previous day (“day 1”)). AM=morning, PM=evening, cfDNA=cell-free DNA, CK=creatine kinase, TRIMP=training impulse. The following questionnaire items from the supplementary material were selected: 1., 6., and 8. (AM questionnaire); 1., 2., 3., and 5. (PM questionnaire).
